# Effectiveness of the Tier 1 Program of Project P.A.T.H.S.: Findings Based on Three Years of Program Implementation

**DOI:** 10.1100/tsw.2010.122

**Published:** 2010-08-03

**Authors:** Daniel T. L. Shek, Rachel C. F. Sun

**Affiliations:** ^1^ Department of Applied Social Sciences, The Hong Kong Polytechnic University, Hong Kong, China; ^2^ Public Policy Research Institute, The Hong Kong Polytechnic University, Hong Kong, China; ^3^ Department of Sociology, East China Normal University, Shanghai, China; ^4^ Kiang Wu Nursing College of Macau, Macau, China; ^5^ Faculty of Education, University of Hong Kong, Hong Kong, China

**Keywords:** adolescents, positive youth development, randomized group trial, P.A.T.H.S., objective outcome evaluation

## Abstract

The Tier 1 Program of the Project P.A.T.H.S. (Positive Adolescent Training through Holistic Social Programmes) is a positive youth development program implemented in school settings, utilizing a curricular-based approach. In the third year of the Full Implementation Phase, 19 experimental schools (n = 3,170 students) and 24 control schools (n = 3,808 students) participated in a randomized group trial. Utilizing the six-wave longitudinal data, ANCOVA, and linear mixed models controlling for differences between the two groups in terms of Wave 1 pretest scores, personal variables, and random effects of schools, it was revealed that participants in the experimental schools showed significantly better development than did participants in the control schools at post-test (Wave 6) based on different indicators of positive youth development derived from the Chinese Positive Youth Development Scale and other measures. Students in the experimental schools also displayed a lower level of intention to engage in problem behavior and better school adjustment than did students in the control schools. Similarly, differences between experimental participants who perceived the program to be beneficial and control participants were found.

### INTRODUCTION

A survey of the literature and research studies shows that there are worrying trends and phenomena related to the development of adolescents in Hong Kong, such as mental health problems[1], abuse of psychotropic substances[2], adolescent suicide[3], school violence[4], compensated dating[5], and drop in family solidarity[6]. A recent study by the Narcotics Division of the Government of the Hong Kong Special Administrative Region showed that the lifetime prevalence of substance abuse in secondary school students has increased to 3.4%[7]. There are also research studies showing that drug abuse[8] and pathological Internet use[9] among young people in Hong Kong are worsening. Although research studies in the West showed that adolescent drug prevention and positive youth development programs are effective means to reduce adolescent developmental problems, there are very few systematic and multiyear positive youth development programs in Hong Kong. Even if such programs exist, they commonly deal with isolated problems and issues in adolescent development (i.e., deficits-oriented programs) and they are relatively short term in nature. Furthermore, systematic and long-term evaluation of the available programs does not exist.

To promote holistic development among adolescents in Hong Kong, The Hong Kong Jockey Club Charities Trust approved HK$400 million to launch a project entitled "P.A.T.H.S. to Adulthood : A Jockey Club Youth Enhancement Scheme" from 2005 to 2009. The word "P.A.T.H.S." denotes **P**ositive **A**dolescent **T**raining through **H**olistic **S**ocial Programmes. As there were positive results for the initial phase of the study[10,11], the Trust approved an additional HK$350 million to sustain the implementation of the project for another 3 years from 2009 to 2012.

To promote positive youth development, a curricular-based program (10- or 20-h Tier 1 Program per grade) was developed for junior secondary school students (i.e., Grades 7–9). The design of the program and constructs included in the program were based on a thorough and systematic review of the scientific literature. In particular, focus was put on the work of Catalano et al.[12] who found that only 25 out of 75 positive youth development programs in North America under review were successful. In these successful programs, 15 positive youth development constructs were identified as the common core components, including promotion of bonding, cultivation of resilience, promotion of social competence, promotion of emotional competence, promotion of cognitive competence, promotion of behavioral competence, promotion of moral competence, cultivation of self-determination, promotion of spirituality, development of self-efficacy, development of a clear and positive identity, promotion of beliefs in the future, provision of recognition for positive behavior, provision of opportunities for prosocial involvement, and fostering prosocial norms.

One unique characteristic of the Project P.A.T.H.S. is that it includes systematic evaluation of the program. Utilizing the principle of triangulation, various evaluation strategies have been used to assess the Tier 1 Program, including objective outcome evaluation, subjective outcome evaluation, process evaluation, interim evaluation, qualitative evaluation based on focus groups, case studies, individual interviews, weekly diaries, and repertory grid technique[13,14,15]. Generally speaking, triangulation of the available evaluation findings shows that different stakeholders had positive views about the Tier 1 Program and they perceived the program to be beneficial to the development of the program participants. Most importantly, the findings suggest that the Project is effective in promoting positive youth development among Chinese adolescents in Hong Kong.

From the perspective of postpositivism, objective outcome evaluation that adopts experimental designs is regarded as the "gold standard" for the evaluation of social intervention programs. Based on a one-group pre/post-test design, Shek[16] concluded that the participants who joined the Experimental Implementation Phase (Grade 7) showed positive changes in several domains of positive youth development after joining the program. Based on a randomized group trial using the data collected at Grade 7 in the Full Implementation Phase (i.e., Wave 1 and 2 data), Shek and associates[17] found that compared with the control group participants, experimental group participants showed greater positive changes in psychosocial competencies and global positive youth development. Utilizing the first four waves of data collected at Grades 7 and 8 in the Full Implementation Phase, statistical findings based on generalized linear models (GLM), linear mixed models (LMM), and individual growth curve models (IGC) also suggested that the experimental subjects performed better than the control subjects[18,19].

The purpose of this study is to examine the effectiveness of the Tier 1 Program of the Project P.A.T.H.S. based on objective outcome evaluation. As the findings reported in the previous studies were limited to Grades 7 and 8 only, there is a need to examine the effectiveness of the Project P.A.T.H.S. over a longer period of time. In this paper, based on Wave 1–6 data collected in the first 3 years (i.e., Grades 7–9 in the Full Implementation Phase), differences between participants in the experimental and control groups with reference to the Wave 1 data (pretest baseline data) and the Wave 6 data (post-test) are reported. In addition, as previous findings showed that roughly one-fifth of the participants did not find the program to be helpful[16], it would be insightful to examine differences between those experimental participants who found the program to be beneficial and the control participants. The general hypothesis is that participants in the experimental group (particularly those perceiving the program to be effective) should perform better than the control participants.

## METHODS

### Participants and Procedures

Shek and associates[17] described the procedures and criteria for recruiting the initial 24 experimental schools (one school dropped out after pretest) and 24 control schools in Year 1, during which Wave 1 and 2 data were collected from Grade 7 students. In Year 2, Wave 3 and 4 data were collected from the same cohort promoted to Grade 8, with 20 experimental schools and 24 control schools. In Year 3, Wave 5 and 6 data were collected from the same cohort promoted to Grade 9, with 19 experimental schools and 24 control schools. The number of students who joined the experimental and control groups in Years 1–3 and the number of completed questionnaires collected can be seen in Table 1.

**TABLE 1**
**Number of Participants and Completed Questionnaires Collected Across 3 Years**

| | Year 1—2006/07 (Waves 1 and 2) | | | Year 2—2007/08 (Waves 3 and 4) | | | Year 3—2008/09 (Waves 5 and 6) | | |
|---|---|---|---|---|---|---|---|---|---|
| | **Exp.** | **Cont.** | **Total** | **Exp.** | **Cont.** | **Total** | **Exp.** | **Cont.** | **Total** |
| Pretest questionnaire collected | 4,121 | 3,854 | 7,975 | 3,290 | 3,861 | 7,151 | 3,170 | 3,808 | 6,978 |
| Pretest questionnaire available for matching (collected with completed unique code) | 4,049 | 3,797 | 7,846 | 3,277 | 3,848 | 7,125 | 3,169 | 3,807 | 6,976 |
| Post-test questionnaire collected | 3,913 | 3,770 | 7,683 | 3,047 | 3,764 | 6,811 | 3,105 | 3,857 | 6,962 |
| Post-test questionnaire available for matching (collected with completed unique code) | 3,880 | 3,730 | 7,610 | 3,047 | 3,763 | 6,810 | 3,104 | 3,856 | 6,960 |
| Successfully matched | 3,443 (50.3%) | 3,405 (50.0%) | 6,848 (100%) | 2,854 (45.0%) | 3,503 (55.0%) | 6,357 (100%) | 2,934 (45.0%) | 3,598 (55.1%) | 6,532 (100%) |

*Note:* The number (percentage) of the successfully matched cases across waves was 4,712 (100%): experimental group, 2,081 (44.2%); control group, 2,631 (55.8%).

At the pre- and post-tests in Years 1–3, the purpose of the study was mentioned and confidentiality of the data collected was repeatedly emphasized to all students in attendance on the day of testing. Parental and student consent had been obtained prior to data collection. All participants responded to all scales in the questionnaire in a self-administration format. Adequate time was provided for the participants to complete the questionnaire. A trained research assistant was present throughout the administration process.

### Instruments

Consistent with the procedures used in Years 1 and 2, participants were invited to respond to a questionnaire containing different measures of youth development at pretest (i.e., before the program began) and post-test (i.e., after the program ended) in Year 3. The following measures were used. The reliability of the scales across the six waves can be seen in Table 2.

**TABLE 2**
**Internal Consistency and Mean Interitem Correlations for All Variables**

| | Wave 1 | | Wave 2 | | Wave 3 | | Wave 4 | | Wave 5 | | Wave 6 | |
|---|---|---|---|---|---|---|---|---|---|---|---|---|
| | α | Mean[a] | α | Mean[a] | α | Mean[a] | α | Mean[a] | α | Mean[a] | α | Mean[a] |
| CPYDS | 0.97 | 0.32 | 0.98 | 0.34 | 0.98 | 0.37 | 0.98 | 0.36 | 0.98 | 0.38 | 0.98 | 0.37 |
| BO | 0.83 | 0.45 | 0.85 | 0.49 | 0.86 | 0.51 | 0.88 | 0.54 | 0.88 | 0.55 | 0.88 | 0.55 |
| RE | 0.82 | 0.44 | 0.86 | 0.50 | 0.88 | 0.54 | 0.88 | 0.55 | 0.89 | 0.56 | 0.88 | 0.55 |
| SC | 0.83 | 0.42 | 0.86 | 0.47 | 0.87 | 0.51 | 0.87 | 0.50 | 0.89 | 0.53 | 0.88 | 0.52 |
| PB | 0.76 | 0.44 | 0.80 | 0.51 | 0.83 | 0.55 | 0.83 | 0.56 | 0.85 | 0.58 | 0.84 | 0.58 |
| EC | 0.83 | 0.44 | 0.85 | 0.48 | 0.86 | 0.51 | 0.86 | 0.51 | 0.87 | 0.52 | 0.86 | 0.51 |
| CC | 0.84 | 0.47 | 0.86 | 0.52 | 0.87 | 0.54 | 0.88 | 0.54 | 0.88 | 0.56 | 0.88 | 0.55 |
| BC | 0.76 | 0.38 | 0.80 | 0.44 | 0.82 | 0.47 | 0.82 | 0.48 | 0.83 | 0.49 | 0.83 | 0.50 |
| MC | 0.78 | 0.37 | 0.79 | 0.39 | 0.81 | 0.42 | 0.80 | 0.41 | 0.82 | 0.44 | 0.82 | 0.43 |
| SD | 0.76 | 0.40 | 0.80 | 0.44 | 0.82 | 0.48 | 0.81 | 0.47 | 0.82 | 0.47 | 0.82 | 0.48 |
| SE | 0.50 | 0.34 | 0.56 | 0.39 | 0.58 | 0.41 | 0.59 | 0.42 | 0.61 | 0.43 | 0.61 | 0.44 |
| SI | 0.84 | 0.43 | 0.85 | 0.45 | 0.87 | 0.48 | 0.86 | 0.47 | 0.87 | 0.48 | 0.87 | 0.49 |
| BF | 0.82 | 0.61 | 0.83 | 0.62 | 0.84 | 0.64 | 0.84 | 0.65 | 0.85 | 0.66 | 0.84 | 0.65 |
| PI | 0.83 | 0.49 | 0.83 | 0.50 | 0.86 | 0.55 | 0.85 | 0.52 | 0.86 | 0.55 | 0.86 | 0.54 |
| PN | 0.77 | 0.40 | 0.80 | 0.45 | 0.81 | 0.46 | 0.81 | 0.46 | 0.81 | 0.46 | 0.81 | 0.47 |
| SP | 0.88 | 0.51 | 0.89 | 0.56 | 0.91 | 0.60 | 0.91 | 0.60 | 0.92 | 0.62 | 0.91 | 0.62 |
| CBC | 0.85 | 0.66 | 0.87 | 0.69 | 0.88 | 0.71 | 0.88 | 0.71 | 0.88 | 0.72 | 0.89 | 0.72 |
| PA | 0.79 | 0.65 | 0.77 | 0.62 | 0.79 | 0.66 | 0.78 | 0.64 | 0.79 | 0.66 | 0.77 | 0.63 |
| GPYDQ | 0.89 | 0.52 | 0.89 | 0.53 | 0.90 | 0.55 | 0.90 | 0.54 | 0.90 | 0.57 | 0.90 | 0.55 |
| PID | 0.83 | 0.72 | 0.84 | 0.73 | 0.85 | 0.75 | 0.85 | 0.74 | 0.86 | 0.76 | 0.86 | 0.76 |
| KEY15 | 0.88 | 0.32 | 0.89 | 0.35 | 0.90 | 0.38 | 0.90 | 0.37 | 0.90 | 0.39 | 0.90 | 0.38 |
| LS | 0.81 | 0.49 | 0.84 | 0.54 | 0.85 | 0.55 | 0.86 | 0.57 | 0.86 | 0.58 | 0.87 | 0.59 |
| TH | 0.91 | 0.33 | 0.90 | 0.33 | 0.90 | 0.32 | 0.89 | 0.30 | 0.88 | 0.30 | 0.88 | 0.30 |
| BI | 0.76 | 0.47 | 0.78 | 0.47 | 0.79 | 0.49 | 0.78 | 0.46 | 0.79 | 0.47 | 0.79 | 0.46 |
| SA | 0.70 | 0.44 | 0.72 | 0.46 | 0.72 | 0.46 | 0.73 | 0.47 | 0.73 | 0.47 | 0.74 | 0.48 |

[a] Mean interitem correlation.

Note: CPYDS: Chinese Positive Youth Development Scale (total score); BO: bonding; RE: resilience; SC: social competence; PB: recognition for positive behavior; EC: emotional competence; CC: cognitive competence; BC: behavioral competence; MC: moral competence; SD: self-determination; SE: self-efficacy; SI: clear and positive identity; BF: beliefs in the future; PI: prosocial involvement; PN: prosocial norms; SP: spirituality; CBC: cognitive-behavioral competencies second-order factor; PA: prosocial attributes second-order factor; GPYDQ: general positive youth development qualities second-order factor; PID: positive identity second-order factor.; KEY15: indicator based on 15 key items; LS: life satisfaction; TH: thriving; BI: behavioral intention to engage in problem behavior; SA: school adjustment.

#### *Chinese Positive Youth Development Scale (CPYDS)*

The scale was developed and validated by Shek and colleagues[20]. Based on the analyses conducted in Year 1[17], the item composition of the 15 subscales of the CPYDS includes Bonding Subscale (BO, six items), Resilience Subscale (RE, six items), Social Competence Subscale (SC, seven items), Emotional Competence Subscale (EC, six items), Cognitive Competence Subscale (CC, six items), Behavioral Competence Subscale (BC, modified five items), Moral Competence Subscale (MC, six items), Self-Determination Subscale (SD, five items), Self-Efficacy Subscale (SE, modified two items), Beliefs in the Future Subscale (BF, modified three items), Clear and Positive Identity Subscale (SI, seven items), Spirituality Subscale (SP, seven items), Prosocial Involvement Subscale (PI, five items), Prosocial Norms Subscale (PN, five items) and Recognition for Positive Behavior Subscale (PB, four items).

Several composite indices based on the above measures were also formed to give a more meaningful picture of the intervention outcomes[20,21]. First, total scale score (CPYDS) was used as a global indicator. Second, based on confirmatory factor analyses, Shek and Ma[21] showed that there were four second-order factors derived from the CPYDS, including cognitive-behavioral competencies second-order factor (CBC), prosocial attributes second-order factor (PA), general positive youth development qualities second-order factor (GPYDQ), and positive identity second-order factor (PID). Third, based on conceptual analyses of the items, one key item was derived from 15 subscales that resulted in a 15-item key measure (KEY15). A higher score indicates a higher level of positive youth development in this study.

#### *Life Satisfaction Scale (LS)*

The Satisfaction with Life Scale was designed by Diener et al.[22] to assess one's own global judgment of one's quality of life. The Chinese version of this scale was translated by the first author and good psychometric properties of the scale have been found[23]. The scale comprised five items, assessing on a six-point response format ranging from "strongly disagree" to "strongly agree". A higher LS scale score indicates a higher level of life satisfaction in this study.

#### *Thriving Scale (TH)*

According to Lerner et al.[24], a person would be regarded as "thriving" if "he or she was involved across time in such healthy, positive relations with his or her community" and on the path to "idealized personhood"[25]. The Search Institute[26] proposed that there are eight thriving indicators that can be used to assess the thriving process. These include success in school (gets almost straight As on report card), helps others (helps friends or neighbors for 1 h or more per week), values diversity (places high importance on getting to know people of other racial/ethnic groups), maintains good health (pays attention to healthy nutrition and exercise), demonstrates leadership (has been a leader of a group or organization in the last 12 months), resists danger (avoids doing things that are dangerous), delays gratification (saves money for something specific rather than spending it all right away), and overcomes adversity (does not give up when things get difficult). Based on this literature, 22 items were developed to assess the concept of thriving on a six-point response format ranging from "strongly disagree" to "strongly agree". A higher scale score indicates a higher level of thriving in this study.

#### *Behavioral Intention Scale (BI)*

Four items were used to assess the intention of the participants to engage in drinking, substance abuse, sexual behavior, and gambling in the next 2 years. For instance, the respondents were asked to assess his/her intention to gamble in the next 2 years with reference to a question ("from now on, will you engage in gambling activities in the next 2 years?"). There are four response options ("absolutely will not", "probably will not", "probably will", and "absolutely will"), with a higher scale score indicating a higher level of intention to engage in problem behavior in this study.

#### *School Adjustment Measures (SA)*

Three items were used to assess the school adjustment of the participants. The first item assessed a respondent's perception of his/her academic performance when compared with schoolmates in the same grade. The respondents were asked to rate "best", "better than usual", "ordinary", "worse than usual", or "worst" in this item. The second item assessed the respondent's satisfaction with his/her academic performance using a five-point response format, i.e., "very satisfied", "satisfied", "average", "dissatisfied", and "very dissatisfied". The final item assessed the respondent's perception of his/her conduct, in which the respondents were asked to rate "very good", "good", "average", "poor", or "very poor". Previous research findings showed that these three items and the related scale were temporally stable and valid[27]. Similarly, a higher scale score indicates a higher level of school adjustment in this study.

Besides the composite scores, three additional items were examined in the study. As leadership qualities are ideal attributes to be cultivated for young people, the first item under examination was "I believe I have some leadership qualities" (F15). In addition, as Internet addiction is a growing problem among adolescents, the item "I know self-restraint when I use the computer" (F21) was examined. Finally, as it is important to know whether young people can uphold their moral principles when they earn money, the item "for the sake of earning money, it is not a problem to sacrifice some moral principles" (F13) was examined.

#### *Subjective Outcomes Scale (SOS)*

Twenty items were used to assess the participant's satisfaction with the program and instructor as well as their perceived benefits of the program at post-tests (i.e., Waves 2, 4, and 6). The response options included "strongly disagree", "moderately disagree", "slightly disagree", "slightly agree", "moderately agree" and "strongly agree". Item 20 (SOS-20) of this scale is "overall speaking, the program was beneficial to my development". Further analyses were carried out by selecting those students who found the program to be beneficial based on Item SOS-20 at Wave 2 as the experimental participants.

### Data Analytic Strategies

Allison et al.[28] pointed out that there were four basic strategies when analyzing change data associated with experimental designs. The first strategy was to examine differences between the experimental and control groups at post-test only. As this strategy did not take into account all information, it was not a recommended approach. The second strategy was to conduct a two-way ANOVA (with group and time as the main effect) and examine the interaction effect between group and time. As this approach was often misinterpreted, it was also not recommended. The third strategy was to look at gain scores. However, as the correlation between pre- and post-test scores seldom equals to 1.0, there would be bias in this analysis. The final recommended strategy was to use ANCOVA to compare post-test scores of the experimental and control groups after statistically controlling for pretest scores. In this study, the final strategy based on ANCOVA with Wave 6 outcomes as dependent variables after statistically controlling for the Wave 1 baseline scores was used. Furthermore, as students in this study were recruited from schools, it could be argued that variations in the outcome measures across groups may also be due to variations in the school characteristics across groups. In order to adjust for the random effect of schools when examining the effect of treatment on the outcome variables[29,30], SPSS linear mixed models were used to conduct the related analyses[31].

### RESULTS

Using schools as the units of analysis, results showed that the 19 experimental schools and 24 control schools did not differ in their school characteristics in the aspects of banding (categorization of students'

academic competence), districts, religious affiliation, gender of the students, and source of funding. For the personal characteristics of the participants, results showed that there were no statistically significant differences between the two groups in their sociodemographic background characteristics ($p > 0.05$ in all cases) except age. In short, except that the mean age of the control group was higher than that of the experimental group, the background characteristics of the experimental and control schools were highly comparable at Wave 1.

For the findings based on ANCOVA controlling for pretest scores at Wave 1, results in Table 3 show that there were significant differences between the experimental and control group participants in terms of various indicators, including the global positive youth development, general positive youth development second-order factor, positive identity second-order factor, intention to engage in problem behavior, school adjustment, life satisfaction, and thriving. For the linear mixed models, the hypothesized models were significantly better than the intercept models, with findings based on the hypothesized models generally positive. The findings showed that the experimental group performed better than the control group in terms of the composite of 15 positive youth development indicators, positive identity subscales, positive identity second-order factor, intention to engage in problem behavior, school adjustment, life satisfaction, and school adjustment indicators after controlling for pretest scores and age as well as adjusting for the random effect of schools. In particular, findings showed that students in the experimental schools were less likely to give up moral principles for the sake of earning money and they had better control when using the computer.

**TABLE 3**
**Differences between the Experimental Group (Joined the Tier 1 Program Only) and Control Group based on the Different Indicators Derived from the CPYDS**

| Global Indicator | Estimated Marginal Mean (Control Group) | Estimated Marginal Mean (Experimental Group) | *F* Value |
|---|---|---|---|
| Findings Based on ANCOVA | | | |
| CPYDS | 4.508 | 4.542 | 3.89, $p < 0.05$ |
| GPYDQ | 4.541 | 4.577 | 3.93, $p < 0.05$ |
| PID | 4.242 | 4.314 | 8.82, $p < 0.005$ |
| BI | 1.530 | 1.445 | 17.78, $p < 0.0001$ |
| SA | 2.988 | 3.058 | 10.80, $p < 0.002$ |
| LS | 3.808 | 3.904 | 9.72, $p < 0.005$ |
| TH | 4.331 | 4.366 | 4.58, $p < 0.05$ |
| Linear Mixed Model Findings Adjusting for the Random Effect of Schools | | | |
| KEY15 | 4.432 | 4.481 | 3.20, $p < 0.05$ (one-tailed) |
| PID | 4.242 | 4.318 | 5.62, $p < 0.05$ |
| BI | 1.532 | 1.461 | 5.46, $p < 0.05$ |
| SA | 2.989 | 3.061 | 4.89, $p < 0.05$ |
| LS | 3.816 | 3.912 | 3.58, $p < 0.05$ (one-tailed) |
| SI | 4.147 | 4.228 | 6.60, $p < 0.02$ |
| F13 | 2.719 | 2.880 | 4.24, $p < 0.05$ |
| F21 | 3.884 | 4.053 | 12.43, $p < 0.002$ |

*Note*: CPYDS: Chinese Positive Youth Development Scale; GPYDQ: general positive youth development qualities second-order factor; PID: positive identity second-order factor; BI: behavioral intention to engage in problem behavior; SA: school adjustment; LS: life satisfaction; TH: thriving; KEY15: indicator based on 15 key items; SI: clear and positive identity; F13: “For the sake of earning money, it is not a problem to sacrifice some moral principles”; F21: “I know self-restraint when I use the computer”.

Further analyses based on experimental participants who found the program to be beneficial (responding in the direction of agreement to item SOS-20 in Wave 2) vs. control participants similarly showed that the experimental participants generally performed better than the control participants in terms of the global and composite positive youth development indicators, all four second-order factors, behavioral intention, school adjustment, life satisfaction, and thriving. In particular, the experimental participants had a higher perceived level of leadership qualities and self-restraint when using computer (see Table 4).

**TABLE 4**
**Differences between the Experimental Group (Joined the Tier 1 Program Only and Perceived the Program to be Beneficial to Their Own Development) and Control Group based on the Different Indicators Derived from the CPYDS**

| Global Indicator | Estimated Marginal Mean (Control Group) | Estimated Marginal Mean (Experimental Group) | *F* Value |
|---|---|---|---|
| Findings Based on ANCOVA | | | |
| CPYDS | 4.517 | 4.589 | 15.90, $p < 0.0001$ |
| CBC | 4.607 | 4.664 | 8.73, $p < 0.005$ |
| PA | 4.515 | 4.576 | 7.87, $p < 0.01$ |
| GPYDQ | 4.551 | 4.624 | 15.20, $p < 0.0001$ |
| PID | 4.250 | 4.366 | 20.26, $p < 0.0001$ |
| BI | 1.527 | 1.443 | 19.92, $p < 0.0001$ |
| SA | 2.992 | 3.091 | 18.74, $p < 0.0001$ |
| LS | 3.815 | 3.956 | 18.59, $p < 0.0001$ |
| TH | 4.337 | 4.401 | 14.42, $p < 0.0001$ |
| Linear Mixed Model Findings Adjusting for the Random Effect of Schools | | | |
| KEY15 | 4.439 | 4.527 | 9.35, $p < 0.005$ |
| BI | 1.529 | 1.449 | 6.95, $p < 0.05$ |
| SA | 2.992 | 3.096 | 8.40, $p < 0.01$ |
| LS | 3.821 | 3.962 | 7.94, $p < 0.01$ |
| TH | 4.343 | 4.401 | 4.89, $p < 0.05$ |
| F15 | 4.100 | 4.228 | 7.61, $p < 0.01$ |
| F21 | 3.889 | 4.107 | 17.16, $p < 0.001$ |

*Note*: CPYDS: Chinese Positive Youth Development Scale; CBC: cognitive-behavioral competencies second-order factor; PA: prosocial attributes second-order factor; GPYDQ: general positive youth development qualities second-order factor; PID: positive identity second-order factor; KEY15: indicator based on 15 key items; BI: behavioral intention to engage in problem behavior; SA: school adjustment; LS: life satisfaction; TH: thriving; F15: "I believe I have some leadership qualities"; F21: "I know self-restraint when I use the computer".

### DISCUSSION

The purpose of this paper is to report objective outcome evaluation findings regarding the effectiveness of a positive youth development program (Project P.A.T.H.S.) in Hong Kong. There are several unique features of the study. First, as there are very few objective outcome evaluation studies utilizing longitudinal data, the present study with six-wave data is a pioneer attempt in the Chinese culture. Second, a validated measure (Chinese Positive Youth Development Scale), with different global measures of positive youth development, was used. In particular, the identification of the second-order factors provides meaningful objective outcome measures to assess the impact of the program. Third, in addition to the Chinese Positive Youth Development Scale, other objective outcome measures, including thriving and life satisfaction, were used. Fourth, both Secondary 1 and 3 data (i.e., Wave 1 data at pretest and Wave 6 data at post-test, respectively) were used in the statistical analyses. Fifth, both ANCOVA and linear mixed models were used to analyze the data based on the postpositivism perspective. Finally, this is the first known scientific study adopting a randomized group trial design using data spanning over 3 years to evaluate a positive youth development program based on a curricular approach in the Chinese communities.

Compared with participants in the control group, participants in the experimental schools performed better in terms of different indicators of positive youth development, thriving, life satisfaction, school adjustment, and had lower intention to engage in problem behavior. In particular, the findings revealed that experimental participants performed better than the control participants in different areas of psychosocial competencies, such as general positive youth development qualities and positive identity, which are crucial ingredients in adolescents' positive and holistic development. Further analyses based on the experimental participants who found the program to be beneficial to their development only (i.e., response to SOS-20 in the positive direction in Wave 2) showed similar, but stronger, results. In addition to the previous findings generated from the objective outcome evaluation based on two- and four-wave data[17,18,19], the present findings based on six-wave data showed that the effect of the program was positive across the three junior secondary school years. In other words, both short- and long-term program benefits to the program participants were realized.

Moreover, there are three aspects of the findings that deserve our attention. First, students in the experimental group had a lower intention to engage in problem behavior as compared to the control group students. This finding is important as it suggests that the program helps to reduce problem behavior in junior secondary school students. Second, the findings showed that the experimental group had higher levels of life satisfaction than the control group. According to Sun and Shek[32], life satisfaction mediates the relationship between positive youth development and adolescent problem behavior. As such, the higher levels of life satisfaction in the experimental subjects could be regarded as a protective factor against problem behavior engagement. As there are few theoretical accounts and research findings on the relationships among positive youth development, life satisfaction, and adolescent problem behavior, it is suggested that further studies in this area, particularly with reference to the utilization of longitudinal data, should be conducted. Third, compared to the control participants, students in the experimental group perceived themselves as having higher levels of leadership qualities, self-restraint when using the computer, and a stronger tendency to uphold moral principles when facing decisions involving money. These findings suggested that the program enhances students' self-perception, self-discipline, and behavioral and moral competencies. All these, in fact, are pertinent human strengths against the gradual worsening of adolescent mental health problems that may lead to indulging in pathological Internet use, drug abuse, compensated dating, and even committing suicide as a way out.

In short, the present objective outcome evaluation study adopting a randomized group trial design lends support to the effectiveness of the Tier 1 Program of the Project P.A.T.H.S. in enhancing students' psychosocial competencies, which is in line with the findings generated from various evaluation strategies, including subjective outcome evaluation, process evaluation, interim evaluation, and qualitative evaluation based on focus groups, case studies, individual interviews, and weekly diaries[10,11,13,14,15]. Following the principle of triangulation, the present study provides scientific longitudinal findings that help to complete a jigsaw puzzle to understand how the Tier 1 Program was beneficial to the program participants.

Although the present findings provide evidence illustrating the program effectiveness of the Project P.A.T.H.S. using longitudinal findings, there are several limitations of the study. First, the effect size associated with the significant findings was on the low side. However, the effect size could be regarded as remarkable as six-wave data spanning over 3 years were involved. Second, as only 3-year data were involved in the program, a relatively immediate effect of the program was revealed. Obviously, it is important to evaluate the long-term effect of the program based on 4- or 5-year longitudinal data. Third, while ANCOVA and linear mixed modes are commonly used to analyze effectiveness of intervention programs, analyses based on growth curves should be used to examine the differences in development changes between experimental and control participants. Despite these limitations, the present study is a ground-breaking scientific study showing the positive impact of the Tier 1 Program of the Project P.A.T.H.S. on the holistic development of Chinese adolescents in Hong Kong.

### ACKNOWLEDGMENTS

The authorship of this paper is equally shared by the two authors. The preparation for this paper and the Project P.A.T.H.S. were financially supported by The Hong Kong Jockey Club Charities Trust.
